# Fractures of the Maxilla

**Published:** 1895-04

**Authors:** P. L. Haight

**Affiliations:** Little Falls, N. Y.


ARTICLE V.

### FRACTURES OF THE MAXILLA.

BY DR. P. L. HAIGHT, LITTLE FALLS, N. Y.

The symptoms are: Crepitus, pain on opening and closing the mouth, swelling and inflammation, inability to masticate and usually a marked irregularity of the teeth. One of the great difficulties in treating these fractures is the fact that, as a rule, the patient will not come for relief till from one to three day's after their accident. They know they have pain, but do not attribute it to a fractured jaw. This is especially so with intoxicated persons, as they usually receive their accident in fighting or from a fall.

Severe fractures of the superior maxilla may occur, and recovery sure. I cite, for instance, the case of Prof. Agnew, of Philadelphia. The patient, a young boy, had been caught between the bumpers of two railroad cars. The whole face had been disjointed, the superior bones badly crushed, and the inferior broken in four places. The upper united; the lower only becoming slightly necrosed, and requiring to be removed. The quick recovery of the superior maxilla was due to the perfect vascularity of the parts.

Fractures of the inferior maxilla may occur at any point, but most frequently near the mental foramen. Should the condyle be fractured it will usually occur at its neck, and this, I think, is the fracture most to be dreaded.

The treatment of these fractures is simple. Bring the parts into perfect apposition, and retain firmly till ossification is complete. This requires from two to four weeks, all greatly depending on the age and condition of the patient. Before taking an impression, it is always well to made a thorough examination for splinters of bone, etc. Should there be any fractured teeth present, or diseased roots, remove them, as their retention might interfere with a perfect union. All exposed pulps or teeth which might possibly ache should be devitalized. The impression should be taken in wax or impression compound. I do not believe in the use of plaster in these cases, for two reasons:

1st. The removal of the impression is painful.

2d. It is too good, for if tin foil is used on the cast, the splint, when vulcanized, will be found to fit the teeth too closely, and too much force will be required to get it in place on the teeth.

It is as well to remove the impression material before it becomes stiff. If it is done carefully and immediately plunged in cold water the impression will be sufficiently perfect.

It was my fortune soon after entering practice to be confronted by a fracture of the inferior maxilla. It was a simple fracture, nearly involving the mental foramen and extending from the body of the bone up to a point directly between the first and second bicuspid. As it occurred the evening before, the face was very much swollen. I decided to set the fracture without reducing the inflammation and swelling. With the help of an assistant I obtained and maintained the exact articulation of the apposing bones. The articulation was maintained by the aid of a piece of soaked boot leather (four by two and one-half inches) sprinkled with plaster of Paris to avoid slipping. This leather, bent in the form of a V, was held by the assistant, who, at the same time, brought pressure to bear through the length of the bone by placing the fleshy part of the thumb over the symphysis and the second finger over the angle. In this way the bone was supported and held in apposition from three points. I then proceeded to get an impression of the coronal surfaces of the teeth as far forward as the lateral incisor and as far back as the second molar. With very soft wax I took an impression of about half the length of the teeth, allowing this to be the distance between the gum margin and the coronal surface.

A model of this I immediately half-flasked. Then, after building wax on the teeth a trifle thicker than I wished the splint, I whole flasked. The flask being separated and the wax removed, I painted the teeth with chlora-percha. This greatly assisted me in packing the rubber about the teeth: Obtaining the proper amount of rubber I reflasked, and closed as rapidly as possible. After vulcanizing, the splint was trimmed down as thin as was consistent with strength and perfect rigidity. My splint was then ready to be inserted in the mouth. I commenced by tying well-waxed silk ligatures around the necks of the teeth to be covered by the splint. The splint being pressed over the crowns of the teeth while the broken bone was in perfect articulation. I found that without other aid it had a tendency to hold the broken parts together firmly. To be sure, I took up the ends of the ligature and tied firmly with a double knot. After applying iodoform gauze to the wound on the cheek, and bandaging only tight enough to hold the dressing, I dismissed the patient. Two weeks from that day the splint was removed. The union was perfect.

To obtain the best and quickest results in fractures of the maxillary bones, tight bandages should always be avoided. If it can be avoided, do not bandage at all. The free and untrammeled supply of blood to the injured part is one of the secrets of success. Since this case I have had two others, both of which were treated in essentially the same manner. During my senior year in college, a friend and I were coasting on our wheels in Fairmount Park. In some way he received a hard tumble, striking his chin and fractured the inferior maxilla at the symphysis. The line of fracture extended up between the central incisors. He was kind enough to allow me the practice. This case I held with the band and screw appliance, making bands to fit snugly over the cuspids. I then soldered to them small tubes of German silver of half an inch in length. These tubes being placed in exact alignment to the teeth. A piece of wire accurately fitting these tubes was bent at right angles at one end and screw cut at the other end. The bands were cemented to place and allowed to thoroughly harden. The wire was then inserted in place in the tubes, and the fractured ends drawn together by tightening the nut on the screw.

This appliance was worn thirty days with little discomfort. An antiseptic wash such as listerin or peroxid of hydrogen should be used by the patient two or three times a day.—*Items of Interest.*

